# Association between perceived chewing ability and oral health-related quality of life in partially dentate patients

**DOI:** 10.1186/1477-7525-8-118

**Published:** 2010-10-19

**Authors:** Mika Inukai, Mike T John, Yoshimasa Igarashi, Kazuyoshi Baba

**Affiliations:** 1 Department of Removable Partial Denture Prosthodontics, Graduate School, Tokyo Medical and Dental University, 1-5-45 Yushima, Bunkyo-ku, Tokyo 113-8549, Japan; 2 Department of Diagnostic and Biological Sciences, University of Minnesota School of Dentistry, 6-320 Moos Tower, 515 Delaware Street SE, Minneapolis, MN 55455 USA; 3 Department of Prosthodontics, School of Dentistry, Showa University, 2-1-1 Kitazenzoku, Ohta-ku, Tokyo 145-8515, Japan; 4 Prosthodontics, New York University College of Dentistry, 345 E, 24th street, New York, NY 10010 USA

## Abstract

**Background:**

One of the most immediate and important functional consequences of many oral disorders is a reduction in chewing ability. The ability to chew is not only an important dimension of oral health, but is increasingly recognized as being associated with general health status. Whether perceived chewing ability and oral health-related quality of life (OHRQoL) are correlated to a similar degree in patient populations has been less investigated. The aim of this study was to examine whether perceived chewing ability was related to OHRQoL in partially dentate patients.

**Methods:**

Consecutive partially dentate patients (N = 489) without signs or symptoms of acute oral disease at Tokyo Medical and Dental University's Prosthodontic Clinic participated in the study (mean age 63.0 ± 11.5, 71.2% female). A 20-item chewing function questionnaire (score range 0 to 20) was used to assess perceived chewing ability, with higher scores indicating better chewing ability. The 14-item Oral Health Impact Profile-Japanese version (OHIP-J14, score range 0 to 56) was used to measure OHRQoL, with higher scores indicating poorer OHRQoL. A Pearson correlation coefficient was calculated to assess the correlation between the two questionnaire summary scores. A linear regression analysis was used to describe how perceived chewing ability scores were related to OHRQoL scores.

**Results:**

The mean chewing function score was 12.1 ± 4.8 units. The mean OHIP-J14 summary score was 13.0 ± 9.1 units. Perceived chewing ability and OHRQoL were significantly correlated (Pearson correlation coefficient: -0.46, 95% confidence interval [CI]: -0.52 to -0.38), indicating that higher chewing ability was correlated with lower OHIP-J14 summary scores (p < 0.001), which indicate better OHRQoL. A 1.0-unit increase in chewing function scores was related to a decrease of 0.87 OHIP-J14 units (95% CI: -1.0 to -0.72, p < 0.001). The correlation between perceived chewing ability and OHRQoL was not substantially influenced by age and number of teeth, but by gender, years of schooling, treatment demand and denture status.

**Conclusion:**

Patients' perception of their chewing ability was substantially related to their OHRQoL.

## Background

One of the most immediate and important functional consequences of many oral disorders is a reduction in chewing ability [1]. The ability to chew is not only an important dimension of oral health [2], but is increasingly recognized as being associated with general health status, because the ability to chew food may affect dietary choices and nutritional intake and may therefore have consequences for general health [3-6].

Chewing problems are common in middle-aged to elderly people. For example, the Florida dental care study found that 23% of participants aged 45 and over who retained at least one tooth had difficulty chewing one or more foods, and 15% were dissatisfied with their ability to chew [7,8]. Other surveys of elderly people have found that one-third of participants had trouble chewing or biting some foods, and this proportion rose to as high as three-fourths in edentulous elderly individuals [9-11].

Impaired chewing ability is perceived as a serious oral health impairment, and has been found to be related to many other oral health problems when assessed with broad concepts such as oral health-related quality of life (OHRQoL). For example, several studies have shown a relationship between self-assessed oral function and OHRQoL [12-16]. Locker *et al*. [13] reported that OHRQoL, as measured by both the 14-item Oral Health Impact Profile (OHIP-14) and the 12-item General Oral Health Assessment Index, discriminated between participants with and without a self-perceived chewing problem in residents of a geriatric care center. Brennan *et al*. [16] reported that the chewing ability index was significantly associated with OHIP-14 scores in the general population.

Whether perceived chewing ability and OHRQoL are correlated to a similar degree in patient populations has been less investigated. It can be expected that both the level of perceived chewing ability and the level of OHRQoL are more impaired in patients. Therefore, the correlation between the two concepts may differ in patients compared with that of the general population. Koshino *et al*. [17] demonstrated a significant association between the levels of chewing ability and OHRQoL impairments, but they exclusively investigated patients with either maxillofacial prostheses and/or complete dentures. No study has examined partially dentate patients, a large population where the study of the relationship between chewing ability and OHRQoL would be informative as to how patients with varying degrees of tooth loss, and therefore varying degrees of chewing ability, perceive their oral health as measured by OHRQoL. In particular, the correlation between perceived chewing ability and OHRQoL may be of clinical relevance, because chewing problems are often the major reason for impaired perceived oral health, which results in treatment demand [18].

It was the aim of this study to investigate the correlation between perceived chewing ability and oral health-related quality of life in partially dentate patients.

## Methods

### Participants and setting

During a three-week study period (June-July 2007), 507 consecutive partially dentate patients without signs or symptoms of acute oral disease at Tokyo Medical and Dental University's Prosthodontic Clinic were enrolled in this study. Almost all patients (N = 496, 98%) participated in the study and provided written informed consent. This study was conducted with approval from the ethics committee of Tokyo Medical and Dental University. (Approval number: #135, December 3, 2005)

The number and location of missing teeth for each participant were recorded. Teeth restored by either implant-supported dentures or fixed partial dentures were not counted as missing teeth and teeth (root) covered with an overdenture were counted as missing teeth. Based on this information, the number of remaining teeth was counted. In addition, the presence of removable partial dentures was recorded.

### Perceived chewing ability measurement

Chewing ability was evaluated by a chewing function questionnaire [19]. This instrument contains 20 food items selected from 100 common Japanese foods. Participants were asked whether it was easy ("1") or difficult ("0") to chew each food. Item responses were combined, resulting in a 0 to 20 summary score that was called the "chewing function score," where higher scores indicate better chewing ability.

Internal consistency of chewing function scores reached a "satisfactory" level [20], with a Cronbach's alpha of 0.90. Test-retest reliability was investigated in a previous study in the same patient population and was considered "fair to good" according to guidelines [21], with an intraclass correlation coefficient based on a one-way analysis of variance of 0.69 (95% CI: 0.56-0.82) for the chewing function score [22].

### Oral health-related quality of life measurement

Oral health-related quality of life (OHRQoL) was measured by the 14-item version of the Japanese Oral Health Impact Profile (OHIP-J14) [23], which characterizes the seven domains (functional limitation, physical pain, psychological discomfort, physical disability, psychological disability, social disability, and handicap) of the original OHIP [18] through the use of two items for each domain. For each of the 14 OHIP questions, participants were asked how frequently they had experienced the impact of that item in the preceding month using a Likert-like scale coded 4 = very often, 3 = fairly often, 2 = occasionally, 1 = hardly ever, and 0 = never. Consistent with the recommended recall period for the Japanese OHIP version [24], 1 month was chosen as frame of reference which provides similar results to the 12-months recall period of the original English-language OHIP according to two studies [25,26]. The OHIP-J14 summary score ranged from 0 to 56, with higher OHIP scores indicating poorer OHRQoL.

Internal consistency (Cronbach's alpha) for the OHIP-J14 was 0.94 and was considered "satisfactory."[20] OHIP-J14 summary score test-retest reliability assessed in a previous study [23] in the same patient population and measured with the intraclass correlation coefficient was 0.73 (95% CI: 0.57-0.88). According to guidelines, this was considered to be "fair to good" [21].

### Data analysis

Seven participants were excluded from the analysis, because there were missing data in either OHIP-J14 or a chewing function questionnaire for those individuals. The data for the remaining 489 participants were analyzed.

Pearson correlation coefficients were calculated to assess the correlation of the OHIP-J14 summary scores and chewing function scores. The magnitude of the correlation was judged according to Cohen [27], with correlations >0.5 considered "large," correlations >0.3 considered "medium," and correlations >0.1 considered "small." In addition, a linear regression analysis was performed, with the OHIP-J14 summary score as the dependent variable and the chewing function score as the independent variable.

Additional Pearson correlation analyses were performed by age, gender, years of schooling, number of teeth, treatment demands (fix/no denture or maintenance group, or needs replacement of new denture) and presence of denture (fix/no denture, complete denture/overdenture; CD/OD in either/both jaws, Kennedy class 1 removable partial denture (RPD) in either/both jaws, Kennedy class 4 RPD in either/both jaws and the other RPDs in either one jaw or both jaws) as major characteristics of physical oral health. Age and number of teeth were split at the variable median into two groups for analyses.

Spearman rank correlation coefficients were calculated for each OHIP-J14 item and chewing function scores.

## Results

### Characteristics of the study population

The mean age of the participants was 63.0 ± 11.5 (range from 19 to 90 years old) and 71.2% were female. The mean number of remaining teeth was 18.3 ± 8.3 (range from 0 to 28 excluding third molars, teeth restored by either implant-supported dentures or fixed partial dentures were not counted as missing teeth and teeth covered with an overdenture were counted as missing teeth.). The majority of the patients (N = 384, 78.5%) had either complete dentures or removable partial dentures in either one jaw or both jaws. Patients had more upper than lower dentures (Table 1). Of the patients with dentures, 199 (51.8%) patients came to the clinic to replace their current dentures.

**Table 1:** Patients' dentures status in both jaws

Jaw	Patients with different denture status						*All patients*
	**No removable denture**	**Removable partial denture**				**Complete Denture Overdenture**	
		**according to Kennedy classification**					
				
		**I**	**II**	**III**	**IV**		
	
				**N (%)**			

**upper**	210 (42.9)	84 (17.2)	74 (15.1)	32 (6.5)	19 (3.9)	70 (14.3)	***489 (100.0)***
**lower**	235 (48.1)	99 (20.2)	94 (19.2)	25 (5.1)	2 (0.4)	34 (7.0)	***489 (100.0)***

### Impaired perceived chewing ability and oral health-related quality of life in partially dentate patients

The mean chewing function score of study participants was 12.1 ± 4.8, with a range of 1 to 20 units. The mean OHIP-J14 summary score was 13.0 ± 9.1, with a range of 0 to 46 units. When participants were divided into "poor" and "good" perceived chewing ability based on the median chewing function score (12.0 units), a significant difference in the proportions of women in the two perceived chewing ability categories was not observed (poor chewing: 73.0% women; good chewing: 69.5%; p > 0.05, Chi-squared test), but age differences were present (poor chewing: 64.5 ± 10.6 years; good chewing: 61.6 ± 12.1 years; t-test, p < 0.01). Participants with a poor perceived chewing ability also had significantly higher OHIP-J14 scores, i.e., they reported more OHRQoL problems than patients with a good chewing ability (poor chewing: 16.6 ± 9.2 OHIP-J14 units; good chewing: 9.5 ± 7.8 OHIP-J14 units; t-test, p < 0.001).

### Correlation between perceived chewing ability and oral health-related quality of life in partially dentate patients

The chewing function score and the OHIP-J14 summary score were substantially correlated (Pearson correlation coefficient: -0.46, 95% CI: -0.52 to -0.38), indicating that better chewing ability was associated with better OHRQoL (R^2 ^= 0.21, p < 0.001). The magnitude of the correlation coefficient was "large." In a regression analysis, a 1.0-unit increase in chewing function score was related to -0.87 OHIP-J14 units (that is, a less impaired OHRQoL; 95% CI: -1.0 to -0.72, p < 0.001).

Effects of gender, age, years of schooling, number of remaining teeth, treatment demand, presence of denture, or Kennedy classification on the association between perceived chewing ability and OHRQoL are summarized in Table 2. None of the correlations was small (0.3≤absolute value of the correlation coefficient). In participants who were male, had more years of schooling, who needed replacement of new denture, wore a CD/OD in either jaw/both jaws, had a Kennedy class I RPD in either jaw/both jaws or a Kennedy class IV in either jaw/both jaws, the correlation was "large" (0.5< absolute value of the correlation coefficient). The smallest correlation coefficient in terms of absolute value was observed for patients with no removable dentures. The largest coefficient was observed for Class IV RPDS; however, the sample size was small for this group of patients.

**Table 2:** Pearson correlation coefficients with 95% confidence interval (95% CI) between perceived chewing ability and oral health-related quality of life for groups of participants stratified by gender, age, years of schooling, number of teeth and presence of denture as indicated.

Variable		n	Correlation coefficient	95% CI
Gender	Male	**141**	**-0.60**	**-0.70 to -0.48**
	Female	**348**	**-0.40**	**-0.49 to -0.31**

Age^1^	<65 years	**252**	**-0.45**	**-0.55 to -0.35**
	≥65 years	**237**	**-0.47**	**-0.56 to -0.36**

Years of schooling	High school education	256*	**-0.40**	**-0.49 to -0.29**
	>High school education	222*	**-0.53**	**-0.62 to -0.43**

Number of teeth^1^	<21	**247**	**-0.47**	**-0.56 to -0.36**
	≥21	**242**	**-0.43**	**-0.53 to -0.32**

Treatment demands	Fix/no denture or RPD maintenance group	**290**	**-0.41**	**-0.50 to -0.30**
	Needs replacement of new denture	**199**	**-0.51**	**-0.61 to -0.40**

Presence of denture(s) and Kennedy classification in RPDs	Fix/no denture	**140**	**-0.38**	**-0.51 to -0.23**
	Class I RPD in either/both jaws	**127**	**-0.51**	**-0.62 to -0.36**
	Class IV RPD in either/both jaws	**12**	**-0.68**	**-0.90 to -0.17**
	Other RPD in either/both jaws	**126**	**-0.43**	**-0.56 to -0.27**
	CD/OD in either/both jaws	**84**	**-0.52**	**-0.66 to -0.35**

^1^Age and number of teeth were split at the variable median

*Some participants refused to answer

When OHIP-J14 items were individually investigated, every item was statistically significantly correlated with the chewing function score (Table 3). The magnitude of the correlations was mostly "medium." The highest correlation was observed for the item, "Have you found it uncomfortable to eat any foods because of problems with your teeth, mouth or dentures?" and the lowest correlation was observed for the item, "Have you been a bit irritable with other people because of problems with your teeth, mouth or dentures?"

**Table 3:** Spearman's correlation coefficients between OHIP-J14 items and chewing function score

OHIP-J14 item^1^			Spearman's rho*	Proportion of "often" or "very often"(%)
functional limitation	1	Trouble pronouncing any words	-0.38	8.38
	2	Sense of taste has worsened	-0.35	6.13
Physical pain	3	Had painful aching in your mouth	-0.31	5.32
	4	Uncomfortable to eat any foods	**-0.43**	7.98

Psychological discomfort	5	Been self conscious	-0.31	11.25
	6	Felt tense	-0.26	4.50

Physical disability	7	Diet has been unsatisfactory	-0.42	4.29
	8	Had to interrupt meals	-0.37	3.07

Psychological disability	9	Difficult to relax	-0.33	3.89
	10	Been a bit embarrassed	-0.33	4.29

Social disability	11	Been a bit irritable with other people	**-0.17**	2.45
	12	Had difficulty doing your usual jobs	-0.26	2.86

Handicap	13	Felt that life in general was less satisfying	-0.31	4.70
	14	Been totally unable to function	-0.27	2.04

* All coefficients p < 0.001

^1 ^Full questionnaire is shown in the appendix

## Discussion

This study demonstrated that individuals' perception of chewing ability is substantially related to oral health-related quality of life in partially dentate patients. More specifically, higher chewing function scores were associated with lower OHIP-J14 summary scores, reflecting that better perceived chewing ability is associated with better OHRQoL. This correlation has been observed previously among older nonpatient populations. Using the Oral Impacts on Daily Performance instrument, Kida *et al*. [28] showed that older adults in nonpatient populations with reduced posterior occlusion were four times more likely to have problems with chewing all food, and twice as likely to report any impairment of daily performance, than their counterparts with intact posterior dentition. Brennan *et al*. [16] also reported a significant association between chewing ability and OHRQoL as measured by OHIP-14 in a population-based sample (random sample, n = 879, age range 45-54). Oral conditions such as infected or sore gums, loose teeth, toothache pain, and fewer functional tooth units have been reported to be associated with onset of chewing difficulty [29]. Our results are in line with this study, because our participants were sampled at a prosthodontic clinic where a majority of them had oral health problems related to tooth loss or dentures. Therefore, based on evidence from different settings and populations, chewing ability seems to have a consistently significant impact on OHRQoL.

It was expected that chewing ability would be related to specific oral health impacts that are directly related to eating, such as "uncomfortable to eat any foods," "diet has been unsatisfactory," and "had to interrupt meals." In our study, the chewing function score was indeed significantly correlated with these three OHIP items, and we observed the highest correlations between chewing function scores and OHIP items for these items, except for a similarly high correlation observed for the item "trouble pronouncing any words." However, the chewing function score was also significantly correlated with all other OHIP items, including psychological dimensions such as "difficult to relax" and "been a bit embarrassed." This finding suggests that chewing difficulty has the potential to have direct or indirect (i.e., because of the correlation with other oral problems) impacts on psychological and social dimensions of oral health. It has been suggested that such effects may be mediated through limitation of food choice and enjoyment of meals and diet [16].

The number of teeth as the major physical characteristic of oral health has previously been reported to impact both chewing ability and OHRQoL in prosthodontic patients [23]. However, in the current study, when correlations between perceived chewing ability and OHRQoL were separately calculated for two populations of participants based on the number of teeth, the correlation between both constructs basically remained unchanged. This result suggests that correlation between perceived chewing ability and OHRQoL is not due to the number of teeth a patient has - a finding which is consistent with the study by Brennan *et al*. [16]. However, when calculated in groups of patients with different denture status, correlations differed more. The findings are exploratory because of the small number of subjects in the groups and the number of analyses performed.

Interestingly, the correlation between perceived chewing ability and OHRQoL did not change much across the two age strata we examined, although age has been associated with chewing ability [30] and OHRQoL [31,32] in previous studies. On the other hand, we observed that the correlation between both constructs was different in men and women and in two categories of years of schooling, with the male patients and those patients with higher years of schooling having the stronger correlations. Although the reasons for these differences were not further explored in the present study, these findings suggest that nonclinical characteristics influence how patients' perceived impaired chewing ability is related to overall perceived oral health, as measured with the concept of oral health-related quality of life.

## Conclusions

Patients' perception of their chewing ability was significantly related to their OHRQoL.

The relationship between perceived chewing ability and oral health-related quality of life status in partially dentate patients attending a prosthodontic clinic is significant, and this relationship is likely influenced by denture status and nonclinical characteristics. Therefore, perceived chewing ability appears to be an important component of perceived oral health.

## Competing interests

The authors declare that they have no competing interests.

## Authors' contributions

MI carried out the outcome studies, participated in the sequence alignment, performed statistical analyses, and drafted the manuscript. MI carried out the data collection. YI participated in the sequence alignment. MTJ participated in the design of the study and the statistical analyses. KB conceived of the study, and participated in its design and coordination. All authors were involved in the manuscript preparation and approved the final manuscript.

## Appendix

OHIP-J14

1. Have you had trouble pronouncing any words because of problems with your teeth, mouth or dentures?

2. Have you felt that your sense of taste has worsened because of problems with your teeth, mouth or dentures?

3. Have you had painful aching in your mouth?

4. Have you found it uncomfortable to eat any foods because of problems with your teeth, mouth or dentures?

5. Have you been self conscious because of your teeth, mouth or dentures?

6. Have you felt tense because of problems with your teeth, mouth or dentures?

7. Has your diet been unsatisfactory because of problems with your teeth, mouth or dentures?

8. Have you had to interrupt meals because of problems with your teeth, mouth or dentures?

9. Have you found it difficult to relax because of problems with your teeth, mouth or dentures?

10. Have you been a bit embarrassed because of problems with your teeth, mouth or dentures?

11. Have you been a bit irritable with other people because of problems with your teeth, mouth or dentures?

12. Have you had difficulty doing your usual jobs because of problems with your teeth, mouth or dentures?

13. Have you felt that life in general was less satisfying because of problems with your teeth, mouth or dentures?

14. Have you been totally unable to function because of problems with your teeth, mouth or dentures?
